# Supplementary material for: The effects of Parkinson’s disease, music training, and dance training on beat perception and production abilities
Source: PLoS One. 2022 Mar 8;17(3):e0264587. doi: 10.1371/journal.pone.0264587 (PMC8903281; doi:10.1371/journal.pone.0264587)
Supplement: S1 Fig — Note: The order of the musical excerpts was randomized. (PDF) [file pone.0264587.s001.pdf]

Song no.	Name	Genre	Metre	Tempo	Alteration	Shift Direction	Shift %	Duration (s)
1	Prime rib	rock	4	104	none			14.1
1	Prime rib	rock	4	104	tempo	faster	2	14.1
1	Prime rib	rock	4	104	phase	ahead	10	14.1
2	Switchblade	rock	4	159	phase	ahead	17.5	12.9
3	Psychedelic space	rock	3	143	none			15.2
3	Psychedelic space	rock	3	143	tempo	faster	2	15.2
4	Sassy Stomp	jazz	4	115	tempo	slower	2	13.6
4	Sassy Stomp	jazz	4	115	phase	ahead	10	13.6
5	Four handed hedgehog	jazz	4	142	none			15.3
6	Four handed hedgehog	jazz	4	142	tempo	slower	2	15.3
6	Four handed hedgehog	jazz	4	142	phase	ahead	17.5	15.3
7	Freedom of the city	pop/orch	4	132	none			11.7
7	Freedom of the city	pop/orch	4	132	tempo	slower	2	11.7
7	Freedom of the city	pop/orch	4	132	phase	ahead	17.5	11.7
8	For king and country	pop/orch	4	85	tempo	slower	2	10.9
9	Lord Arbinger Waltz	pop/orch	3	165	tempo	faster	2	13
10	Crazy	jazz	3	165	tempo	faster	2	16
practice1	One jump ahead	pop/orch	4	120	phase	ahead	17.5	12
practice2	Roaring twenties	jazz	4	108	tempo	slower	10	13.5
practice3	Never going back again	rock	4	120	none			12
