# Supplementary material for: The effects of Parkinson’s disease, music training, and dance training on beat perception and production abilities
Source: PLoS One. 2022 Mar 8;17(3):e0264587. doi: 10.1371/journal.pone.0264587 (PMC8903281; doi:10.1371/journal.pone.0264587)
Supplement: Attachment — Submitted filename: Response_To_Reviewers.docx [file pone.0264587.s002.docx]

Dear Dr. Sonja Kotz,

Thank you for handling our manuscript, “The effects of Parkinson’s disease, music training, and dance training on beat perception and production abilities,” for possible publication in Plos One. We also thank the reviewers for their careful feedback and helpful comments. We now submit a revised version, with additions about beat production tapping variability. Point-by-point responses to the reviewers’ comments are attached below. Note that the line numbers refer to the Revised Manuscript with Tracked Changes file. We hope that you might find this version to be suitable for publication for the wide readership of Plos One.

Sincerely,

Prisca Hsu, Emily Ready, Jessica Grahn

**Reviewer #1**

**Introduction**

- “**simple timing tasks [8] and more complex beat-based rhythm discrimination tasks [2].” (l65). What are the simple tasks that the authors refer to? Interval-based? It would be helpful to describe briefly the tasks typically used in these experiments here. In the same paragraph, two other types of tasks are introduced (“rhythm discrimination tasks”, l67, and “temporal discrimination task”, l69). Please clarify by giving a short description of the tasks, as very few readers will be familiar with that.**

Indeed, there is room to clarify what is meant by simple and complex here. We have added task clarification in lines 64-66:

Patients are impaired on tapping tasks involving finger tapping to a metronome followed by paced tapping without a metronome [8] and more complex rhythm discrimination tasks that required participants to decipher whether two beat-based rhythms were identical [2].

- **Note that in some studies, the BAT is only the perceptual task ([11], [13], [16] in the article reference list), whereas in others it includes a variety of other tasks ([14] in the article reference list). It would be worth mentioning it to avoid possible confusion.**

Yes, good point. Reference 11,13 and 16 in the original submission, only used the perception component of the BAT and used another, different, synchronization task to measure beat production. We have now noted that these studies only utilized the beat perception component of the BAT.

L137-142: Though beat perception and production are thought to be related, there is evidence that the abilities dissociate [24,25], thus many assessments include both perceptual and production tasks [16,26,27]. The Beat Alignment Test (BAT) is one such assessment [26]. The beat perception task of the BAT has been extensively used in various studies [15,24] and has successfully identified people with impaired beat perception but intact beat production, and vice versa [24,25].

- **The second paragraph of the “Rhythm disturbance in Parkinson’s disease” section does not seem to fit in well with the section. The authors can consider moving it to another subsection.**

We agree that the second paragraph seems more about the relationship between production/perception and the BAT, not on rhythm disturbances in PD and not about the neural underpinnings. We have moved this paragraph to the Study Rationale section.

- **Because the article does not include any brain imaging measures, I suggest not to start by describing the brain areas involved in PD, music training, and dance training in the different sections. Focusing on the behavior is more relevant. Neuronal underpinnings can be briefly mentioned at the end of each paragraph, probably in one sentence, especially because they are not discussed in the rest of the article.**

We agree that the descriptions of anatomical differences between musicians/non-musicians and dancers/non-dancers aren’t relevant to the current study, we have now removed that information.

- **“As the basal ganglia have not been implicated in the neural changes associated with music and dance training” (l126). I would tone down this statement. It may be true that no changes have been directly observed in the basal ganglia, and I trust the authors who have certainly carefully reviewed the literature on this topic. Nevertheless, this absence of observed change in the basal ganglia may have different causes, and the basal ganglia are part of networks that are associated with music and dance training.**

Fair point. We have tempered the sentence accordingly:

L155-159: We hypothesized that music and dance training would correlate with better beat perception and production abilities, while Parkinson’s disease would reduce these abilities relative to controls. The behavioral benefits of music and dance training may still be preserved in Parkinson’s disease. Therefore, Parkinson’s disease patients with previous training may have better abilities than patients without training.

**Methods**

**- Is there a significant difference in age between the older adults and the PD groups?**

The mean age for older adults is 64.5 and Parkinson’s disease patients is 68.2. A Welch two-sample t-test does show a significant difference between mean ages for the two groups (p = .01), but linear models fitting age and beat perception, asynchrony, coefficient of variation and coefficient of deviation indicate no significant effects of age on the dependent variables (all p’s > .05). We believe that the age difference between older adults and Parkinson’s disease patients is therefore not driving any group differences in beat perception or production.

Each graph is showing age of the participant along the x-axis and the relevant dependent variable along the y-axis. These graphs include data points from both healthy older and Parkinson’s groups.

We have added this information to our results section, clarifying that there is a significant difference between the ages of healthy older adults and Parkinson’s patients, but this age difference does not reliably affect in beat perception and production abilities.

L298-317: The mean age for older adults (64.5) significantly differed from that of Parkinson’s disease patients (68.2), as shown by a Welch’s unequal variance two sample t-test (t = -2.58, p = .012). However, linear models fitting age and beat perception, asynchrony, coefficient of variation and coefficient of deviation indicated age did not predict performance on any of the dependent variables in these groups. Therefore, age differences in the range found in the older adults and Parkinson’s disease group do not appear to reliably affect beat perception or production (all p’s > .05).

- **“Six participants who did not complete both beat perception and production tasks of the BAT and participants who did not indicate the years of previous music and dance training experience were excluded from the analyses” (l140-142). Does the final N of participants presented in table 1 include those participants? If so, I suggest to rather present only the participants that were included in the analyses.**

The final N=458 does not include participants who did not complete both beat perception and production tasks or did not indicate years of music/dance training. We have clarified this in the Participants section.

L169-173: Six participants who did not complete both beat perception and production tasks of the BAT and eight participants who did not indicate the years of previous music and dance training experience were excluded from the analyses. The final N=458 only includes participants who completed the BAT and indicated the years of previous music and dance training experience.

- **The initial battery developed by Müllensiefen, Gingras, Musil, & Stewart (2014) that is used in this study contains 18 stimuli. Why did the authors use only 17? I would find it useful to give some precision about the stimuli (e.g., N of trials with phase and period changes, percentage of change etc.) so that the reader does not have to read the article by Müllensiefen et al. to understand the methods. This is particularly important if the number of stimuli differs.**

Indeed, this is correct. The musical stimuli for the BAT were taken from the Beat Alignment Test of the Goldsmiths Musical Sophistication Index (Gold-MSI) v1.0 downloaded from <https://www.gold.ac.uk/music-mind-brain/gold-msi/download/>. This version was recommended to us by the authors of the 2014 study when we contacted them for stimuli, as Version 1.0 of the Gold-MSI is optimized relative to the version reported in Müllensiefen et al., 2014’s study, with 17 items, selected as described in the documentation available at <https://www.gold.ac.uk/music-mind-brain/gold-msi/download/>). We added a table outlining the BAT stimuli as supplementary material, and added a reference to the website and a specific version number of the BAT to clarify exactly which version was used. This also adds relevant information about the number of trials with phase and period shifts, as well as the amounts of those shifts on each trial.

L195-199: Musical stimuli were taken from the Beat Alignment Test of the Goldsmiths Musical Sophistication Index (Gold-MSI) v1.0 [26] downloaded from https://www.gold.ac.uk/music-mind-brain/gold-msi/download/. Version 1.0 of the Gold-MSI is optimized relative to the version reported in Müllensiefen et al., (2014), with 17 items, selected as described in the documentation available at https://www.gold.ac.uk/music-mind-brain/gold-msi/download/ (S1 Table).

L213-216: Phase shifts of the superimposed beeps were adjusted 10% or 17.5% ahead relative to the musical beat. Period shifts of the superimposed beeps were adjusted 2% slower or faster relative to the musical tempo (S1 Table).

- **“low asynchrony scores reflected less variable and more consistent tap times” (l180). I do not think the asynchrony score reflect variability and consistency. It tells how far from the beat the participants tapped; a high score can reflect a very consistent performance in antiphase, for example. None of the two indexes reflect variability; it could be worth using a coefficient of variation of the asynchronies [SD(asynch)/Mean(asynch)], where asynch = Mean(Response - Beat). This index might reveal additional effects that the tempo matching index failed to capture. Notably, I expect that participants with dance and music training are less variable in their tapping performance. For example, a recent study on children with cerebellar anomalies showed that a dance training protocol reduced their variability in a synchronization task (Bégel et al., 2021).**

This is a great suggestion. We have adjusted the interpretation for high/low asynchrony scores and analyzed coefficient of variability scores. The CoV data is now included in all results.


**Results**

**- “When assessing the effects of dance training on beat perception, the null model had a BF10 of 1 (Table 2b), suggesting that the data could not discriminate between the alternative hypothesis from the null hypothesis” (l288-290). This is also the case for the music & group model. Please explain a bit more how you come to this conclusion and what is the difference between the two Null models. This remark applies to the phase matching analyses as well.**

We added a brief explanation of the 3 logical states (null hypothesis supported, null hypothesis rejected, insufficient information to discriminate between the two hypotheses) in relation to BF_10_ values in the “Bayesian analyses” paragraph.

L391-401: The Bayesian ANOVA compares the predictive performance of each model with and without each independent variable and their interactions. The P(M) column is the prior model probability which assumes that all rival models are equally likely to represent the data. The P(M|data) column indicates the probability of each model given the actual data. The BFm column indicates the relative likelihood of each model compared to the average of all other models, and the BF10 column indicates the relative likelihood of each model compared to the null model. Generally, a BF10 < 1/10 provides strong support for the null hypothesis, BF10 > 10 provides strong support for the alternative model, and 1/3 < BF10 < 3 indicates that the data is insufficient to support either hypothesis [29,30]. The strength of the evidence can be quantified based on the Bayes Factor (e.g., BF10 =8 is twice as strong as BF10 = 4 in supporting the alternative hypothesis).

- **Figure 2. Because the PD group was not included in the analyses with dance training, I think their results should not appear in the figure.**
We removed the PD group from the dance figures.

- **Figure 3. It is not clear what the three lines (user, wide, ultrawide) are. Most readers are not familiar with Bayesian statistics. It is important to give more information.**

Thanks for noting this, we realized the sequential aspect of the analysis isn’t relevant to the analysis and have decided to remove the figure entirely.

**Discussion**

- **The absence of difference between the PD and healthy adults groups in beat perception is inconsistent with the results of Benoit et al. (2014). There are interindividual difference in Parkinson’s patients that may explain the contradictory results (Cochen de Cock et al., 2018). I think this should be mentioned in the discussion.**

Thanks for the suggestion. We have referenced and discussed the Benoit et al. (2014) and Cochen et al., (2018) papers in our discussion section.

L499-504: In contrast, our results differed from Benoit et al.’s findings that Parkinson’s patients showed worse timing perception than healthy adults [31]. However, in their study, Parkinson’s patients were tasked to detect misaligned beats in a two-measure music excerpt, as opposed to several seconds of tones overlaid on music in the BAT. This task is less taxing on memory and attention, and thus may not be able to differentiate between healthy adult groups and Parkinson’s patients.

L528-530: Cochen de Cock et al. corroborated these findings by suggesting that cognitive abilities such as attention, executive function and cognitive flexibility could influence beat perception abilities [32].

- **The “Implications” and “Limitations” sections are quite long. Condensing them into one section seems appropriate.**

We have condensed these paragraphs into one section**.**

**Reviewer #2**

**The main limitation is that the information about the training is very limited: we don’t know when the training was and what its intensity and level was. This an important limitation in this study that is discussed in the discussion.**

We agree this is a limitation in our study and have noted that in our discussion section.

L543-544: Years of training is an imprecise quantification of true training effects, as the rigor of different training programs and hours of deliberate practice varies across individuals.

**There is also an important lack of information on Parkinson’s disease. Hoehn and Yahr stage is very insufficient to evaluate PD severity. Disease duration Ldopa Equlivalent daily dose are needed to understand the kind of patients that were explored. These two information should be added.**

Thanks for noting this limitation, we agree that the Hoehn and Yahr scale provides limited information on the PD severity. We unfortunately do not have other information on the PD participants regarding to their disease and have noted this limitation in our discussion section.

L611-615: Hoehn and Yahr stage is also the only indicator of disease severity collected in the study, and future work addressing the impact of these factors across disease stages would benefit from including more specific disease severity information such as disease duration or the levodopa equivalent daily dose and by including participants with moderate to severe stages of Parkinson’s.

**Also the recent reference on rhtythm disturabances in PD and prePD should be added and discussed**

The reference was added to our introduction section.

L73-76: Furthermore, beat perception and production deficits are correlated with idiopathic REM sleep disorder which commonly occurs prior to Parkinson’s disease onset and is often considered a prodromal-Parkinson’s disease symptom [11].
