# Supplementary material for: The effects of Parkinson’s disease, music training, and dance training on beat perception and production abilities
Source: PLoS One. 2022 Mar 8;17(3):e0264587. doi: 10.1371/journal.pone.0264587 (PMC8903281; doi:10.1371/journal.pone.0264587)
Supplement: Attachment — Submitted filename: Response_To_Reviewers_Rev2.docx [file pone.0264587.s003.docx]

Dear Dr. Sonja Kotz,

Thank you for handling our manuscript, “The effects of Parkinson’s disease, music training, and dance training on beat perception and production abilities,” for possible publication in Plos One. We also thank the reviewers for their careful feedback and helpful comments. We now submit a revised version, with further clarifications about our Bayesian analyses. Point-by-point responses to the reviewers’ comments are attached below. Note that the line numbers refer to the Revised Manuscript with Tracked Changes file. We hope that you might find this version to be suitable for publication for the wide readership of Plos One.

Sincerely,

Prisca Hsu, Emily Ready, Jessica Grahn

**Reviewer #1**

**I believe there is still a problem with the sentence “When assessing the effects of dance training on beat perception, the null model had a BF10 of 1 (Table 2b), suggesting that the data could not discriminate between the alternative hypothesis from the null hypothesis”. Description of table 4 is similar. Again, all null models have a BF10 of 1, so how can that be supporting the null hypothesis in itself? I believe the reason is that all other models have a BF10 comprised between 1/3 and 3.**

Thanks for pointing this out. We realize that the description of the BF10 may be confusing to our readers. As noted in L311-317, a BF10 value of 1 falls between 0.33 and 3 and thus indicates inconclusive results that do not clearly support H0 or H1 models. We have rephrased those sentences.

L331-333: When assessing the effects of dance training on beat perception, the null model had a BF_10_ of 1 (Table 2b), suggesting that the data are inconclusive and do not support either null or alternative models.

L367-369: For tempo matching, the Bayesian ANOVAs for both music and dance revealed a BF_10_ = 1 (Tables 4a & 4b). A BF_10_ = 1 value suggests that the data are inconclusive and do not support either null or alternative models.

**I suggest presenting the interpretation for the full range of possible BF10 values: “BF10 < 1/10 provides strong support for the null hypothesis, 1/10 < BF10 < 1/3 provides moderate support for the null hypothesis”, and again 3 < BF10 < 10. It would also be useful to explicitly say that the BF10 value is a ratio that is expressed in decimal in the tables. I had difficulties to understand that.**

This is great suggestion. We have adjusted the Bayesian statistics description and changed the BF10 values to decimal notation in the following lines.

L311-317: As BF10 deviates from 1, support for the null or alternative hypothesis increases. Generally, 0.33 < BF10 < 3 indicates that the data is insufficient to support either null or alternative hypothesis. 0.1 < BF10 < 0.33 provides moderate support for the null hypothesis and 3 < BF10 < 10 provides moderate support for the alternative hypothesis. Finally, a BF10 < 0.1 provides strong support for the null hypothesis and BF10 > 10 provides strong support for the alternative hypothesis. [29,30]. Strength of the evidence can be quantified based on the Bayes Factor (e.g., BF10 =8 is twice as strong as BF10 = 4 in supporting the alternative hypothesis).

**I am not convinced that scientific notation should be used in table 2. Numbers are not that big and scientific notation can be confusing. Three numbers presentations are used for BF10 when presenting the interpretation of the index and the values (ratios, decimals and scientific), which make it very hard to follow.**

Thanks for noting this. We have changed the notation of the numbers in table 2. We have also changed the BF_10_ values to decimal notation in the Bayesian Analyses section.
