# Supplementary material for: The effects of Parkinson’s disease, music training, and dance training on beat perception and production abilities
Source: PLoS One. 2022 Mar 8;17(3):e0264587. doi: 10.1371/journal.pone.0264587 (PMC8903281; doi:10.1371/journal.pone.0264587)
Supplement: Attachment — Submitted filename: Response_To_Reviewers_Rev3.docx [file pone.0264587.s004.docx]

Dear Dr. Sonja Kotz,

Thank you for handling our manuscript, “The effects of Parkinson’s disease, music training, and dance training on beat perception and production abilities,” for possible publication in Plos One. We thank reviewer 1 for their careful feedback and helpful comments. We now submit a revised version, with further clarifications about our Bayesian analyses. We hope that you might find this version to be suitable for publication for the wide readership of Plos One.

Sincerely,

Prisca Hsu, Emily Ready, Jessica Grahn

**Reviewer #1:**

**Thanks a lot for addressing my comments; I think the presentation of the Bayesian analyses is much improved, which is very important as most readers are not familiar with it.
Nevertheless, I still think there is a major mistake with the sentence ‘When assessing the effects of dance training on beat perception, the null model had a BF10 of 1 (Table 2b), suggesting that the data are inconclusive’. I already pointed that in my previous reviews: all null models have a BF10 of 1, included the one presented in table 2a, where the authors say that data are conclusive. I believe that the null model’s BF is always 1. Unless I am missing something, this sentence is wrong and makes the Bayesian results really confusing. The sentence describing the model in the ‘Beat production: tempo matching’ section is also incorrect. Please modify that.**

Thank you for your careful review. You are correct. All BF10 values are relative to the BF10 value of the null model. As such, the BF_10_ value for the null model will always be 1. We realize that our indication of interpreting the BF_10_=1 is confusing and have modified those lines to be clearer about how the BF_10_ values should be interpreted.

L325-327 When assessing the effects of dance training on beat perception, the dance model had a BF10 value of 0.33 (Table 2b), indicating insufficient support for either the null hypothesis or the alternative hypothesis [29,30].

L362-364 For tempo matching, the music model had a BF10 of 0.50 (Table 4a), indicating insufficient support for either the null hypothesis or alternative hypothesis. For dance, BF10 = 0.22 (Table 4b), indicating moderate support for the null hypothesis.
